# Intravenous Immunoglobulin for Management of Non-paraneoplastic Autoimmune Retinopathy

**DOI:** 10.18502/jovr.v15i2.6743

**Published:** 2020-04-06

**Authors:** Sahba Fekri, Masoud Soheilian, Babak Rahimi-Ardabili

**Affiliations:** ^1^ Ophthalmic Research Center, Shahid Beheshti University of Medical Sciences, Tehran, Iran; ^2^ Department of Ophthalmology, Labbafinejad Medical Center, Shahid Beheshti University of Medical Sciences, Tehran, Iran

**Keywords:** Autoimmune Retinopathy, Intravenous Immunoglobulin, Nyctalopia, Photopsia, Retinal Degeneration

## Abstract

**Purpose:**

To report a case of non-paraneoplastic autoimmune retinopathy (npAIR) treated with intravenous immunoglobulin (IVIG).

**Case report:**

A 12-year-old boy presented with progressive visual field loss, nyctalopia, and flashing for three months. He had suffered from common cold two weeks before the onset of these symptoms. On the basis of clinical history and paraclinical findings, he was diagnosed with npAIR, and IVIG without immunosuppressive therapy was started. During the one-year follow-up period after the first course of IVIG, flashing disappeared completely. Visual acuity remained 10/10, but nyctalopia did not improve. Multimodal imaging showed no disease progression.

**Conclusion:**

Although established retinal degenerative changes seem irreversible in npAIR, IVIG may be a suitable choice to control the disease progression.

##  INTRODUCTION

Autoimmune retinopathies (AIRs) are a heterogeneous group of immune-mediated degenerative retinal disorders, caused by circulating antiretinal antibodies (ARAs). They can be paraneoplastic (pAIR) or, more commonly, non-neoplastic (npAIR) characterized by bilateral, often asymmetric, progressive, painless visual acuity or visual field loss over weeks to months with photopsias and scotomas.^[1]^ Depending on the cell type and antigen targeted by ARAs, signs and symptoms may be diverse and overlapping. Insufficient knowledge of the clinical course, prognosis, and treatment of AIRs makes their management challenging. Here, we report a case of npAIR stabilized with intravenous immunoglobulin (IVIG).

##  CASE REPORT

**Figure 1:**
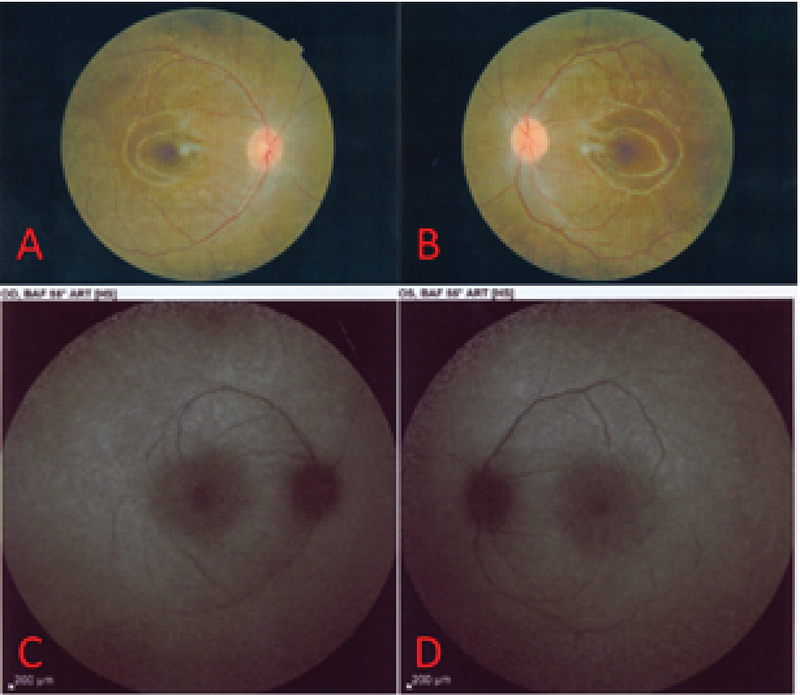
(A & B) Color fundus photograph shows optic disc pallor, vascular attenuation, and diffuse retinal atrophy with mottling of the retinal pigmented epithelium. (C & D) Perifoveal and perivascular hyperautofluorescence is noticeable on fundus autofluorescence imaging.

**Figure 2:**
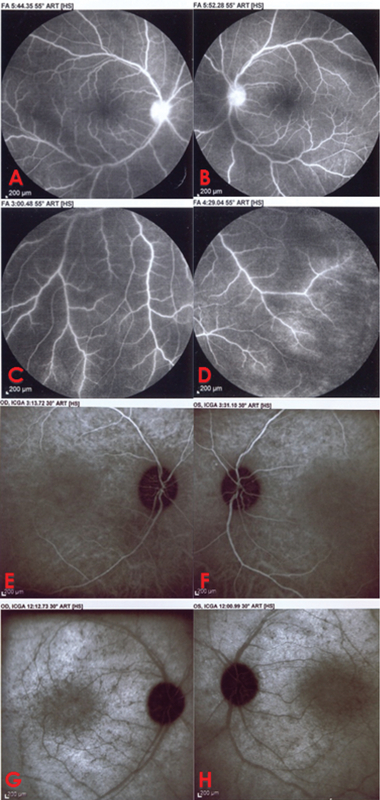
(A & B) Disc hyperfluorescence is seen on fluorescein angiography. (C & D) Mild vascular leakage (almost from veins) is more prominent at retinal mid-periphery on fluorescein angiography. (E–H) Indocyanine green angiography shows multiple hypocyanescent spots in late stages.

**Figure 3:**
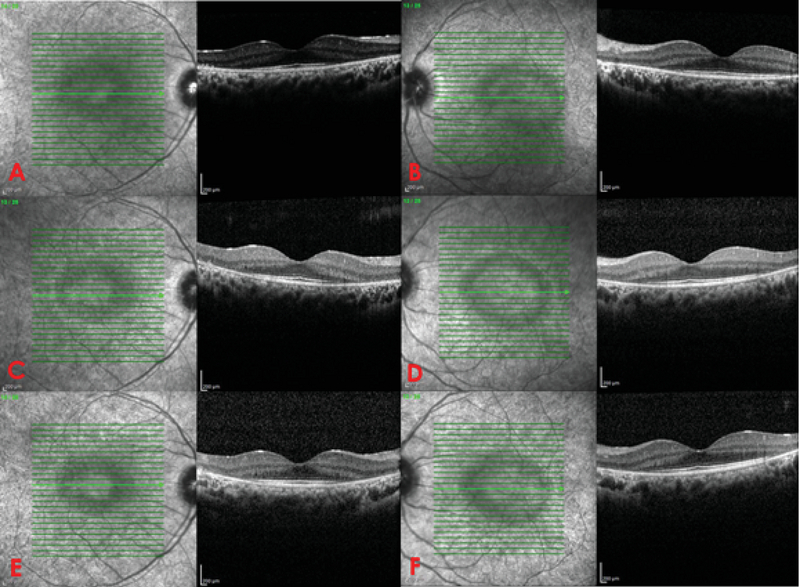
(A & B) Optical coherence tomography demonstrates loss of the photoreceptor layer, disruption of the ellipsoid zone, loss of the external limiting membrane, and thinning of the outer nuclear layer with central sparing at baseline. (C & D) After one-year follow-up and the last course of IVIG therapy, there is no change in retinal structures. (E & F) Six months after discontinuation of IVIG therapy (18-month follow-up), stability of retinal atrophic changes is seen.

**Figure 4:**
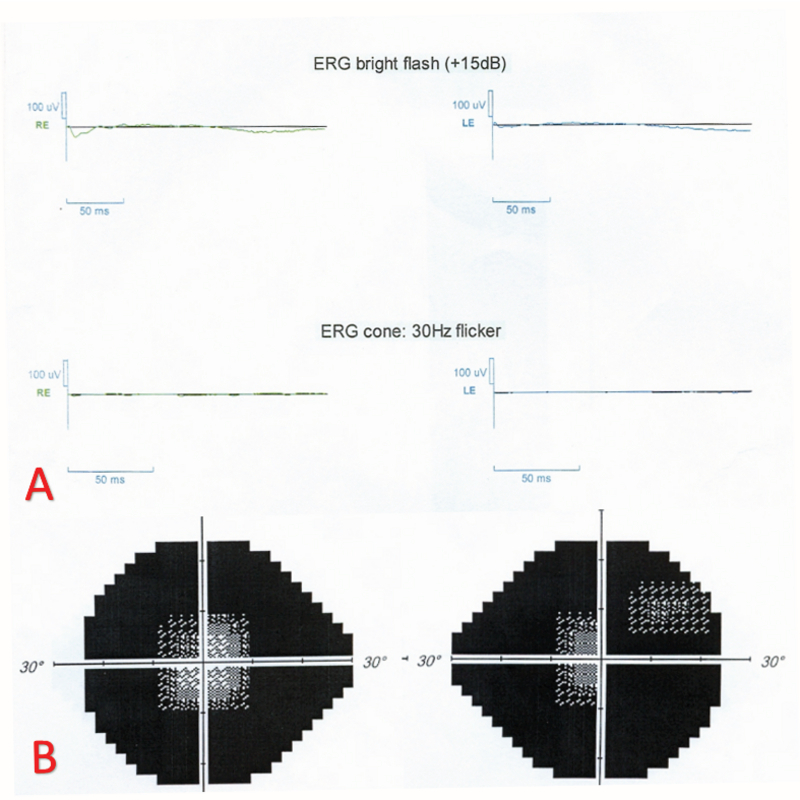
(A) Both photopic and scotopic responses are severely extinguished on full-field electroretinography. (B) The 24-2 Humphrey visual field depicts severe field constriction with foveal sparing.

A 12-year-old boy was referred to our clinic because of progressive visual field loss and photopsia for three months. He was mentally alert and performed well at school. He experienced flashing approximately two weeks after a common cold episode. It rapidly progressed to decreased night vision and visual field defects with difficulty walking downstairs. There was no relevant past medical, surgical, or drug history. The family history was negative for autoimmune and hereditary disorders. Systemic work-ups, including cell count, biochemistry, and liver, kidney, and thyroid function tests were unremarkable. The erythrocyte sedimentation rate and C-reactive protein level were within normal ranges. The patient tested negative for the antinuclear antibody, anti-neutrophil cytoplasmic antibody, rheumatoid factor, complement components 3 and 4, total complement activity, Venereal Disease Research Laboratory test, anti-toxoplasma antibody, hepatitis B surface antigen, hepatitis C virus antibody, and antihuman immunodeficiency virus antibody. No malignancies or rheumatologic disorders were detected. The corrected distance visual acuity was 10/10 OU with normal color vision and no relative afferent pupillary defects. Slit lamp examination showed normal anterior segment and intraocular pressure with no inflammation. Dilated funduscopy revealed optic disc pallor, vascular attenuation, and diffuse retinal atrophy with mottling of the retinal pigmented epithelium [Figure 1]. There were no bone spicules or vitreous cells. Fundus autofluorescence (FAF) imaging revealed perifoveal and perivascular hyperautofluorescence [Figure 1]. Fluorescein angiography (FAG) revealed disc hyperfluorescence with mild vascular leakage [Figure 2]. Late-phase indocyanine green angiography (ICGA) showed multiple hypocyanescent spots [Figure 2]. Optical coherence tomography (OCT) demonstrated loss of the photoreceptor layer, disruption of the ellipsoid zone, and thinning of the outer nuclear layer with central sparing [Figure 3]. Full-field electroretinography (ERG) showed severely extinguished photopic and scotopic responses [Figure 4]. The 24-2 Humphrey visual field confirmed advanced field constriction sparing fixation [Figure 4]. The presumptive diagnosis was npAIR based on clinical and paraclinical findings and disease course. Serologic tests for ARAs were not available in the country, and the patient could not afford genetic testing. Systemic steroids (1 mg/kg) with IVIG (400 mg/kg/day for five days) were started. His parents did not consent to immunosuppressive therapy, therefore IVIG was considered. Systemic steroids were tapered slowly and discontinued after three months, but IVIG was repeated every three months for one year. He received four courses of the IVIG therapy. During the 18-month follow-up period, clinical and paraclinical features of the disease remained stable, with no progression or improvement. Photopsia disappeared completely. Visual acuity was 10/10 OU at all visits, and visual field defects or OCT abnormalities did not progress [Figure 3]. After each injection of IVIG, the patient reported an improvement in the quality of vision lasting for two weeks and then returning to the previous situation; however, we could not assess these changes objectively.

##  DISCUSSION

Autoimmune retinopathy is a rare immune-mediated disorder, triggered by molecular mimicry between retinal proteins and certain antigens that induce immunologic sensitivity in the host.^[2]^ Based on the presumed nature of these antigens and underlying etiology, AIR is categorized into pAIR, which is related to underlying malignancies and the similarity between tumor antigens and retinal proteins, and npAIR, which correlates with the similarity between presumed infectious antigens and retinal proteins. The final outcome of all forms is retinal degeneration. Underlying malignancies should be ruled out before the diagnosis of npAIR is made.^[3]^ Signs and symptoms may be diverse and overlapping, depending on the cell type and antigen targeted by ARAs. Patients usually present with acute or subacute, bilateral vision loss, visual field defects, nyctalopia, scotomas, and photopsia. Funduscopy may be unremarkable or reveal optic disc pallor, vascular attenuation, retinal atrophy, and retinal pigment epithelium mottling with minimal or no vitritis.^[1]^ Therefore, ancillary tests seem necessary to detect retinal degenerative changes. Signs and symptoms of npAIR are non-specific and may overlap with other retinal disorders, including hereditary retinal diseases, other degenerative retinal disorders, particularly retinitis pigmentosa (RP), drug toxicity, and various inflammatory or infectious etiologies.^[4]^ Finally, to establish the diagnosis and rule out other causes, FAG, FAF, ICGA, visual field (VF), ERG, and OCT may be helpful. Khanna et al reported multimodal imaging properties of npAIR,^[5]^ including the characteristic pattern of diffuse or granular, stippled hyperautofluorescence throughout the posterior pole on FAF, peripheral visual field constriction, amplitude reduction under scotopic and photopic conditions on ERG, and attenuations of the outer nuclear layer and ellipsoid zone parafoveally on OCT. Moreover, they suggested a diagnostic algorithm for clinical use according to which pathognomonic findings should be obtained from at least one objective, structural test (SD-OCT and/or FAF) and one objective, functional test (ERG and/or VF, if available). In our patient, results of ancillary tests were compatible with npAIR, and the acute onset of symptoms, clinical course of the disease, and negative history of hereditary retinal disorders ruled out RP. Clinical findings of RP and npAIR overlap, making the definitive diagnosis challenging. Increasing knowledge of AIRs in the past years has created doubt regarding patients who have been followed-up for RP without any family history of RP or typical bone spicules. In a case series, immunosuppressive therapy was started for two patients despite positive family history of RP because they had classic signs of npAIR, and visual function improved during follow-up.^[6]^ ARAs play a pivotal role in the pathogenesis of AIR.^[2]^ The detection of circulating ARAs can confirm the diagnosis in patients with a high suspicion of npAIR based on clinical and paraclinical findings.^[7]^ However, various laboratory techniques help detect ARAs, but lack of a globally accepted technique and poor inter-laboratory concordance make this process challenging.^[1]^ Moreover, ARAs may be found in other inflammatory or degenerative disorders, even in the normal population. Conversely, ARAs may not be found in many patients with AIR.^[8]^ The detection of ARAs in a patient can represent the following: (1) the primary pathogenesis of retinal changes; (2) a normal unrelated finding; (3) an epiphenomenon resulting from the release of retinal antigens by a pathologic process; or (4) a secondary process worsening a pathologic progress. It is difficult in many cases to determine which of the above are operational.^[2]^ Although in the consensus for standardization of diagnostic criteria for AIR,^[7]^ the detection of ARAs was an essential component, because of the aforementioned reasons, lack of ARAs would not rule out the diagnosis. Serologic tests for ARAs were unavailable for our patient.

The management of npAIR is also controversial. There is no global consensus on the management or prognosis of AIRs^[3]^ because most of our knowledge comes from case reports and case series. Suggested approaches include systemic and local corticosteroids, antimetabolites such as mycophenolate mofetil, azathioprine, and T cell inhibitors such as cyclosporine, targeted B-cell therapy such as anti-CD20 monoclonal antibody (rituximab), IVIG, and plasmapheresis.^[1]^ In most cases, visual prognosis is uncertain, but improvement in visual field and visual acuity may occur. Therapy is not effective in patients with widespread retinal degeneration. IVIG consists of human immunoglobulin G obtained from the plasma of healthy donors. Proposed mechanisms of action of IVIG in autoimmune disorders include modulation of cellular immunity by antibody-dependent cytotoxicity, anti-idiotypic antibodies, blockade of immune complex binding to receptors, blockade of cell-to-cell interactions, induction of regulatory T cells, and downregulations of T and B cell activations.^[9]^ IVIG is usually used as a second-line therapy in autoimmune diseases. We used it in this patient because his parents did not consent to immunosuppressive therapy. Guy et al^[10]^ reported three cases of pAIR treated with IVIG. In one patient, visual acuity improved dramatically within 24 h of taking the first dose, but visual field improved two weeks later. In the second patient, there was no improvement, although ARA levels declined. In the third patient, the visual field improved. Treatment was started in early stages of disease for all patients, contrary to our patient who had profound anatomical and functional degenerative retinal changes at presentation. A recent study showed that although the treatment of npAIR did not produce anatomical improvement, it ensured stability of symptoms and prevented involvement of the second eye in unilateral cases.^[5]^ The disappearance of photopsia, subjective improvement in our patient's vision quality, and stabilization of the disease highlight the possible role of IVIG in npAIR management.

##  Financial Support and Sponsorship

None.

##  Conflicts of Interest

There are no conflicts of interest.
